# Genome-wide analysis of peptidase content and expression in a virulent and attenuated *Babesia bovis* strain pair

**DOI:** 10.1016/j.molbiopara.2011.06.005

**Published:** 2011-10

**Authors:** Maria Mesplet, Guy H. Palmer, Monica J. Pedroni, Ignacio Echaide, Monica Florin-Christensen, Leonhard Schnittger, Audrey O.T. Lau

**Affiliations:** a Instituto de Patobiología, CICVyA, INTA-Castelar, Buenos Aires, Argentina; b Consejo Nacional de Investigaciones Científicas y Técnicas (CONICET), Buenos Aires, Argentina; c Program of Genomics, Department of Veterinary Microbiology and Pathology and Paul G. Allen School for Global Animal Health, College of Veterinary Medicine, Washington State University, Pullman, WA 99164-7040, United States; d Estación Experimental Agropecuaria Rafaela, INTA, Argentina

**Keywords:** *Babesia bovis*, Apicomplexans, Peptidases, Transcriptome, Virulence

## Abstract

Identifying virulence determinants in Apicomplexan parasites remains a major gap in knowledge for members within this phylum. We hypothesized that peptidases would segregate with virulence between a virulent parent *Babesia bovis* strain and an attenuated daughter strain derived by rapid *in vivo* passage. Using the complete genome sequence of the virulent T2Bo strain, 66 peptidases were identified and active sites confirmed. The presence, sequence identity and expression levels were tested for each of the 66 peptidases in the virulent parent and attenuated daughter T2Bo strains using whole genome, targeted sequencing approaches and microarrays analyses. Quantitative PCR revealed that there was no significant difference in peptidase expression between the virulent and attenuated strains. We conclude that while peptidases may well play a required role in *B. bovis* pathogenesis, neither loss of peptidase gene content nor reduced gene expression underlies the loss of virulence associated with *in vivo* passage and attenuation.

## Graphical abstract

**Graphical abstract:**

Bovipain-2 (in red), a *Babesia bovis* cysteine protease, is released into the host erythrocyte. Bovipain-2 is expressed in both virulent and attenuated *B. bovis*. DAPI stains the nuclei of two *B. bovis*.

**Figure:**
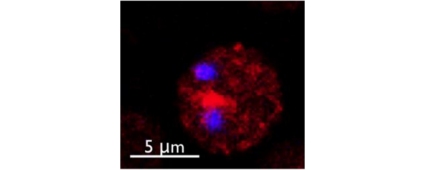

**Highlights:**

► Genome sequence of the virulent T2Bo strain reveals 66 peptidases which constitute 2% of the annotated coding sequences. ► Each of the peptidases identified in the virulent parent was represented in the attenuated daughter strain. ► Microarrays analyses of transcript levels for all 66 peptidase-encoding genes were investigated. ► Our study discusses our findings in regards to peptidase involvement in *in vivo* attenuation of *B. bovis*.

Virulence is a dynamic characteristic of microbial pathogens. Pathways frequently associated with virulence include enhanced invasion, increased replication, triggering of host inflammatory responses, and evasion or suppression of host immunity [1–5]. For the genetically complex pathogens in the Phylum *Apicomplexa*, definitive identification of virulence determinants remains a gap in knowledge—a gap relevant to control of major animal and human diseases. We are addressing this gap using a virulent and attenuated strain pair of *Babesia bovis*. The T2Bo strain is highly virulent in naïve animals and requires chemotherapeutic treatment to prevent severe morbidity and progression to death. In contrast, attenuation of this strain by sequential *in vivo* passage in splenectomized calves generates a daughter strain that is markedly less virulent than the parent T2Bo: duration and peak of parasitemia are significantly less, anemia significantly less severe, and infected animals do not require treatment. The virulent parent T2Bo strain has been sequenced and annotated [6] and the attenuated daughter has been sequenced to 93% coverage by pyrosequencing. Our goal is to use this virulent-attenuated strain pair to identify virulence determinants of babesial parasites and define the pressures that select for virulent versus attenuated parasites in nature.

Proteases and peptidases are enzymes that, in addition to the participation in multiple vital cellular functions in both prokaryotic and eukaryotic organisms, have been identified as virulence factors for Apicomplexan parasites. Plasmepsin 4 is a plasmodial aspartic peptidase that participates in lysosomal hemoglobin digestion. Absence of this peptidase reduces virulence in experimentally infected hosts: cerebral malaria is abrogated and parasites are cleared [7]. In *Theileria* spp., serial subcultivation of *T. annulata in vitro* is associated with the gradual loss of metallopeptidase activity and virulence, illustrating the relationship between peptidase activity and virulence [8]. Specifically for babesial parasites, Wright et al. and Savon et al. proposed that peptidases may be a specific virulent determinant [9,10]. This is functionally supported by the ability of specific cysteine peptidase inhibitors to impair *B. bovis* merozoite development *in vitro* [11].

The present study was designed to test the hypothesis that peptidases are a dynamic virulence determinant that is lost during *in vivo* attenuation of *B. bovis*. *In silico* analysis of the *B. bovis* predicted proteome revealed the presence of 66 putative proteases in the virulent T2Bo strain, which constitutes approximately 2% of the total protein-coding genes in the parasite genome; similar to the 2–3% of total coding sequences that encode peptidases in other completely sequenced Apicomplexan parasites [12–17]. These include 5 aspartic, 18 cysteine, 19 metallo-, 18 serine, and 6 threonine proteases (Table S1). Fig. 1 demonstrates the clade and family distribution. Among the 66 peptidases, 26 were new putative peptidases predicted using the MEROPS database (Table S2), not reported in the original genome annotation [6]. These were cysteine- (*n* = 7), metallo- (*n* = 3), serine- (*n* = 11) and threonine (*n* = 5) peptidases. A search of open reading frames flanking the identified peptidase-encoding genes did not reveal any additional candidates.

Among the 18 putative *B. bovis* cysteine peptidases, five have orthologues in both *Plasmodium falciparum* and *Toxoplasma gondii*: peptidase families C1, C12, C13, C19, and C48 (Table S3). Five of the cysteine peptidases have orthologues only in *P. falciparum* (C1, three of C19, and C44) and C78 has an orthologue only in *T. gondii*. *P. falciparum* and *T. gondii* have seven peptidase orthologues exclusively found between them and not in *B. bovis* (C11, C14, C15, C50, C65, C86 and C88). An expansion of C19 family is observed in *B. bovis* (*n* = 4) and in *P. falciparum* (*n* = 7) whereas *T. gondii* has only one.

Each of the 66 peptidases of the T2Bo virulent parent was aligned with its homologue in the T2Bo attenuated daughter in order to identify differences at the genomic level. Nucleotide and amino acid sequences were identical for 60/66 peptidases. One synonymous single nucleotide polymorphism (snSNP) was observed in BBOV_II005940, a gene that encodes for an intramembrane protease rhomboid 4, a S54 peptidase family (data not shown). Not only was the change synonymous but it also did not occur at the predicted amino acid active site [18] or within the predicted peptidase region. Thus, we conclude that BBOV_II005940 is unlikely responsible for the phenotypic variation between the virulent and attenuated pair.

The remaining five peptidase genes required amplification from both genomes as the genomic data base sequences were incomplete (Table S4). Sequences of 4/5 of these genes revealed no sequence difference at the nucleotide level between the virulent parent and its attenuated derivative. Nucleotide differences were found in BBOV_III009030, an Ufm1-specific peptidase that belongs to a C78 family. Amplification followed by sequence comparison of multiple clones indicated that 11% of those present in the virulent parental strain were identical to the sequence as previously reported [6] while 89% of the sequences consist of 41 non-synonymous SNPs, albeit no deleterious amino acids changes predicted by SIFT software. This is consistent with T2Bo being oligoclonal rather than a true clone. These two subpopulations are referred to as I and II (Fig. S1). Identical distribution of BBOV_III009030 subpopulations I and II (12% and 88%, respectively) were found in the attenuated derivative strain. It is therefore, concluded that two subpopulations of BBOV_III009030 gene of similar proportion in exist in both the virulent parent and attenuated daughter and that this peptidase is unlikely to be responsible for the phenotypic variation. Furthermore, this suggests that the attenuation process does not simply reflect a shift in the proportions of clones within the strain. The overall lack of coding sequence differences for all the peptidases between the virulent and attenuated parasites clearly indicates that loss of virulence by *in vivo* attenuation of virulence is not governed at the genomic level.

Using a microarray based on the genomic sequence of the parental *B. bovis* virulent strain, transcription of the complete 66 peptidase gene repertoire was tested using the virulent and attenuated pair (detailed design and statistical analysis of transcriptome array are provided in supplemental material). All predicted peptidases were transcribed in the virulent T2Bo in the three biological replicate pairs used for array hybridization. A fourth biological replicate strain pair was added for subsequent validation of the array results. Out of 66 peptidases, seven peptidases exhibited significant differential expression levels between virulent and attenuated samples (Table 1). These are BBOV_I000200, _I000540, _II001130 and _III003510 in the attenuated sample and BBOV_I004260, _III000270 and _IV008660 in the virulent sample. The attenuated verses virulence signal ratio (A/V) of these seven peptidases was either >2 or <0.5, which indicates that the fold differences in the hybridization signals based on their transcription are either up-regulated in the attenuated or virulent samples, respectively. However, the differential expression levels of these peptidases between the strain pair were not uniform among all biological replicates; specifically, differential transcription of these peptidases was observed only in biological replicate pair #1 (Table 1). In order to validate the array data, qPCR was performed using the same biological replicate sample pairs (#1–3) as well as a biological replicate sample pair #4. Using BBOV_III004820 that encodes for topoisomerase II to normalize the qPCR assay, cycle thresholds (CT) for the seven peptidases were measured. After normalization, these peptidase expressions were represented as cycle threshold ratios and subsequently pooled (pCT) based on four technical replicates (Fig. S2). Table 1 shows the corresponding pCT ratios of these peptidases. Analyses using a two-tailed unpaired Student's *T*-test with confidence level set at 95%, pCT ratios demonstrate that there were no statistically significant differences of the pCT ratios between the virulent and attenuated parasites for these seven peptidases (Table 1, Fig. S2). The observed fluctuation in transcriptional levels of these seven peptidases appears to have been within the physiological range in a biological system at any given time. Thus, we conclude that, in addition to the remaining 59 peptidase genes, these seven peptidases do not have significantly different levels of transcription between virulent and attenuated *B. bovis*.

Based on our genomic and transcriptional analyses, we reject the hypothesis that *in vivo* attenuation is associated with loss of peptidase function at either the genomic or transcriptional levels. This conclusion is significant as it separates attenuation generated by *in vivo* passage from a requirement for peptidases in virulence. That is, peptidases may well be required for virulence, a proposition supported by multiple studies with a diverse set of Apicomplexan parasites [19], but are not affected by *in vivo* attenuation in the mammalian host. To the degree that *in vivo* attenuation reflects the natural acquisition and loss of virulence among the parasite population during sequential transmission events, these results suggest that while peptidase content and expression are retained, and perhaps required for maintaining the infectious cycle, the dynamic virulence determinants are yet to be uncovered. Identifying these determinants and how they specifically interact with the host and variable host factors such as immunity is a key step to both better understanding and control of virulent Apicomplexan parasites.

## Figures and Tables

**Fig. 1:**
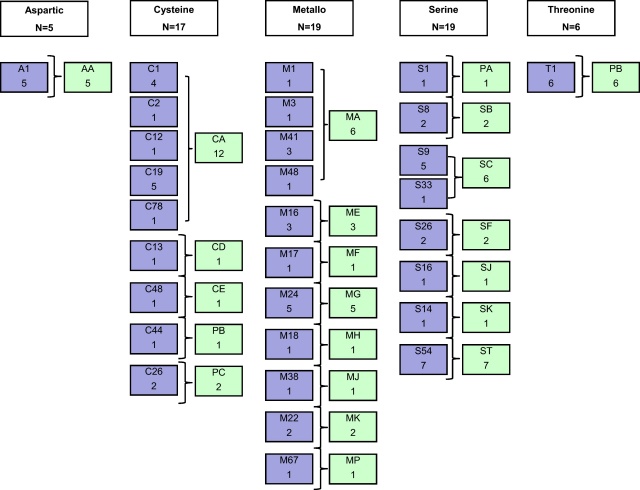
The complete *Babesia bovis* peptidase repertoire. Colored blocks represent different peptidase families. Blue, aspartic; yellow, cysteine, red, metallo; green, serine and orange, threonine. Briefly, putative *B. bovis* peptidases in the genome of *B. bovis* virulent strain T2Bo were identified *in silico* using the batch blast resource of MEROPS website (http://merops.sanger.ac.uk) [20]. Responses with *p* values < 10E^−4^ were analyzed by using BLAST in MEROPS or PIR sites (http://pir.georgetown.edu) [21] to identify the presence of active sites characteristic of protease function. Peptidase regions were determined using MEROPS, PFAM or prosite prediction. The molecular weight and isoelectric point for each putative peptidase was calculated using Vector NTI 8.1. (For interpretation of the references to color in this figure legend, the reader is referred to the web version of the article.)

**Table 1:** Array signal ratio (A_*n*_/V_*n*_) and subsequent transcript levels of seven *Babesia bovis* peptidase in virulent and attenuated strain pair.

Gene ID^a^	Annotation	A_1_/V_1_	A_2_/V_2_	A_3_/V_3_	pCT^b^ ratio_vir_ (±SEM)	pCT^b^ ratio_att_ (±SEM)	Significance *p* < 0.05
	Conserved hypothetical protein	2.15	1.08	1.16	1.00 (±0.03)	1.02 (±0.01)	N
	Dipeptidylpeptidase	2.69	1.84	0.73	1.079 (±0.01)	1.093 (±0.02)	N
	Hypothetical protein	2.02	0.91	0.99	1.153 (±0.03)	1.195 (±0.02)	N
	Eimepsin	2.15	1.23	0.78	1.230 (±0.03)	1.202 (±0.01)	N
BBOV_I004260	Hypothetical protein	0.45	1.41	1.06	1.20 (±0.07)	1.29 (±0.06)	N
BBOV_III000270	Hypothetical protein	0.33	1.15	1.11	1.71 (±0.08)	1.55 (±0.09)	N
BBOV_IV008660	Proteosome catalytic subunit 2	0.49	0.74	0.85	1.03 (±0.05)	1.05 (±0.03)	N

a Blue genes have signal ratios of >2 in at least one biological replicate set (indicating their gene expression levels were upregulated in T2Bo_att) while black genes have signal ratio of <0.5 (indicating their expression levels were upregulated in T2Bo_vir.A, ^2^A/V (attenuated/virulent); A_1_/V_1_, A_2_/V_2_, A_3_/V_3_ indicate the three microarray replicates.

b CT ratio = CT value_gene of interest_/CT value_4820 (house keeping gene)_ for each biological replicate sample set; *p*, pooled values from four biological replicates.
