# Supplementary material for: Genome-wide analysis of peptidase content and expression in a virulent and attenuated Babesia bovis strain pair
Source: Mol Biochem Parasitol. 2011 Oct;179(2-2):111–3. doi: 10.1016/j.molbiopara.2011.06.005 (PMC3167272; doi:10.1016/j.molbiopara.2011.06.005)
Supplement: Supplementary file 1 [file mmc1.doc]

Table S1. *In silico* prediction of *B. bovis* peptidases repertoire. n/d: non determined. Blue shade: aspartic; cream shade, cysteine; grey shade, metallo; yellow shade, serine and orange shade, threonine peptidase species.

| **Gene ID** | **Protein GenBank Accession Number** | **GenBank Designation** | **Family & subfamily** | **Clan** | **Merops Number** | **Merops Peptidase subtype** | **Protein length (aa)** | **MW (KDa)** | **pI** | **Peptidase region** | **Active site residues** | **Metal ligands** | **Chr** | **Locus start** | **Locus end** |
| --- | --- | --- | --- | --- | --- | --- | --- | --- | --- | --- | --- | --- | --- | --- | --- |
|
| BBOV_III003510 | XP_001611483 | eukaryotic aspartyl protease family protein | A01A | AA | MER107413 | eimepsin | 521 | 58.5 | 5.25 | 139-497 | D167,Y213,D358 |  | 3 | 783622 | 785288 |
| BBOV_IV010360 | XP_001610957 | aspartyl protease, putative | A01A | AA | MER107412 | unassigned | 532 | 58.57 | 7.96 | 180-511 | D203,F249,D403 |  | 4 | 1413759 | 1415460 |
| BBOV_IV009660 | XP_001610888 | aspartyl protease, putative | A01A | AA | MER107411 | unassigned | 435 | 48.86 | 4.62 | 27-388 | D58,Y105,D327 |  | 4 | 1269306 | 1271035 |
| BBOV_IV007890 | XP_001610711 | aspartyl protease, putative | A01A | AA | MER107410 | unassigned | 463 | 52.17 | 6.65 | 141-460 | D169,F214,D359 |  | 4 | 885265 | 887572 |
| BBOV_III001640 | XP_001611299 | aspartyl protease family protein | A01B | AA | MER204823 | plasmepsin-5 | 540 | 61.07 | 7.09 | 27-409 | D104,Y169,D332 |  | 3 | 376890 | 378512 |
| BBOV_III010070 | XP_001612131 | papain family cysteine protease containing protein | C01A | CA | MER181456 | papain homologue {{Theileria}-type} | 435 | 49.1 | 5.39 | 217-426 | Q244,C250,H379,N399 |  | 3 | 2166068 | 2167391 |
| BBOV_II000170 | XP_001609546 | cathepsin C precursor, putative | C01A | CA | MER165808 | dipeptidylpeptidase I {{Plasmodium}-type} | 530 | 59.65 | 6.35 | 262-511 | Q279,C285,H454,N478 |  | 2 | 40948 | 42754 |
| BBOV_I000540 | XP_001608716 | preprocathepsin c precursor, putative | C01A | CA | MER165458 | dipeptidylpeptidase I {{Plasmodium}-type} | 546 | 61.34 | 5.88 | 279-530 | Q300,C306,H474,N498 |  | 1 | 140974 | 142827 |
| BBOV_IV007730 | XP_001610695 | cysteine protease 2 | C01A | CA | MER181338 | papain homologue {{Theileria}-type} | 445 | 49.32 | 7 | 235-444 | Q254,C260,H389,N411 |  | 4 | 850162 | 851499 |
| BBOV_III007670 | XP_001611895 | calpain family cysteine protease domain containing protein | C02 | CA | MER099391 | Mername-AA253 peptidase | 894 | 99.56 | 6.89 | 96-399 | Q121,C127,H333,N358 |  | 3 | 1661418 | 1664102 |
| BBOV_III010630 | XP_001612184 | ubiquitin carboxyl-terminal hydrolase, family 1 protein | C12 | CA | MER142065 | unassigned | 275 | 31.04 | 5.37 | 23-266 | Q116,C122,H196,D223 |  | 3 | 2274792 | 2276054 |
| BBOV_I000200 | XP_001608682 | conserved hypothetical protein | C13 | CD | n/d | n/d | 498 | 56.5 | 8.83 | 76-409 | H223,C265 |  | 1 | 70642 | 72176 |
| BBOV_III008630 | XP_001611991 | ubiquitin carboxyl-terminal hydrolase family protein | C19 | CA | MER109765 | unassigned | 496 | 56.04 | 5.12 | 76-477 | N118,C123,H422,D448 |  | 3 | 1863144 | 1864704 |
| BBOV_III006180 | XP_001611747 | ubiquitin carboxyl-terminal hydrolase family protein | C19 | CA | n/d | n/d | 713 | 80.45 | 8.93 | 80-425 | N102,C108,H370,N386 |  | 3 | 1335209 | 1337350 |
| BBOV_III001650 | XP_001611300 | ubiquitin carboxyl-terminal hydrolase family protein | C19 | CA | n/d | n/d | 1073 | 120.99 | 5.75 | 778-1072 | N781,C786,H1015,D1033 |  | 3 | 380116 | 383337 |
| BBOV_IV004350 | XP_001610364 | ubiquitin carboxyl-terminal hydrolase, putative | C19 | CA | n/d | n/d | 790 | 88.35 | 4.9 | 295-788 | N334,C339,H751,N766 |  | 4 | 131993 | 134646 |
| BBOV_IV001730 | XP_001609338 | ubiquitin carboxyl-terminal hydrolase, putative | C19 | CA | n/d | n/d | 1446 | 164.45 | 5.33 | 188-606 | N201,C206,H522,D539 |  | 4 | 392203 | 396585 |
| BBOV_III003590 | XP_001611491 | carbamoyl phosphate synthetase | C26 | PC | MER201193 | unassigned | 1632 | 179.46 | 5.75 | 288-465 | C363,H447 |  | 3 | 796281 | 801299 |
| BBOV_II005340 | XP_001610053 | cytidine triphosphate synthetase, putative | C26 | PC | n/d | n/d | 554 | 61.67 | 6.37 | 349-530 | C383,H509 |  | 2 | 1211190 | 1212961 |
| BBOV_IV000250 | XP_001609193 | glucosamine-fructose-6-phosphate aminotransferase | C44 | PB | MER165603 | unassigned | 723 | 80.59 | 6.02 | 53-295 | C53 |  | 4 | 66439 | 68825 |
| BBOV_II004090 | XP_001609932 | ulp1 protease family, C-terminal catalytic domain containing protein | C48 | CE | MER120078 | unassigned | 390 | 45.63 | 6.19 | 181-360 | H273,D292,Q342,C348 |  | 2 | 949842 | 951014 |
| BBOV_II005540 | XP_001610073 | hypothetical protein | C54 | CA | n/d | n/d | 206 | 23.82 | 7.85 | 35-202 | Y39,C79,D192,H194 |  | 2 | 1253712 | 1254554 |
| BBOV_III009030 | XP_001612028 | protein of unknown function (DUF1671) protein family | C78 | CA | MER109140 | UfSP2 peptidase | 677 | 75.81 | 5.18 | 482-668 | Y493,C505,D630,H632 |  | 3 | 1948134 | 1950468 |
| BBOV_IV005930 | XP_001610522 | aminopeptidase, putative | M01 | MA | MER079999 | alanyl aminopeptidase {bacterial-type} | 846 | 95.94 | 5.61 | 33-458 | E335,Y418 | H334,H338,E357 | 4 | 472477 | 475179 |
| BBOV_III002610 | XP_001611394 | peptidase family M3 containing protein | M03A | MA | MER166075 | unassigned | 507 | 57.06 | 6 | 10-507 | E364 | H363,H367,E392 | 3 | 604427 | 606142 |
| BBOV_IV001260 | XP_001609291 | mitochondrial processing peptidase beta subunit | M16B | ME | MER138288 | mitochondrial processing peptidase beta-subunit | 514 | 57.88 | 5.97 | 71-203 | E112,E182 | H109,H113,E189 | 4 | 288536 | 290283 |
| BBOV_II001130 | XP_001609640 | hypothetical protein | M16B | ME | MER188775 | chloroplast (stromal) processing peptidase | 1138 | 129.02 | 5.36 | 5-210 | E48,E122 | H45,H49,E129 | 2 | 286144 | 289633 |
| BBOV_II004890 | XP_001610012 | peptidase M16 inactive domain containing protein | M16C | ME | MER140236 | unassigned | 1166 | 131.37 | 5.77 | 174-701 | E183,E258 | H180,H184,E295 | 2 | 1138858 | 1142358 |
| BBOV_II004450 | XP_001609968 | leucine aminopeptidase, putative | M17 | MF | MER105931 | unassigned | 525 | 56.6 | 5.17 | 132-520 | K306,R382 | K294,D299,D318,D378,E380 | 2 | 1045409 | 1047164 |
| BBOV_IV011550 | XP_001611075 | aspartyl aminopeptidase , putative | M18 | MH | MER119941 | aspartyl aminopeptidase | 429 | 48.02 | 6.18 | 15-419 | D96,E267 | H94,D221,E268,D310,H404 | 4 | 1668712 | 1670308 |
| BBOV_III009430 | XP_001612067 | glycoprotease family protein | M22 | MK | MER189469 | unassigned | 406 | 45.27 | 6.2 | 101-396 | n/d | H209,H213 | 3 | 2018666 | 2020053 |
| BBOV_II003000 | XP_001609823 | glycoprotease family protein | M22 | MK | MER117006 | Kae1 putative peptidase | 358 | 38.62 | 6.63 | 12-323 | n/d | H117,H121 | 2 | 697281 | 698373 |
| BBOV_III008650 | XP_001611993 | methionine aminopeptidase, putative | M24A | MG | n/d | n/d | 330 | 36.03 | 6.37 | 103-329 | H157 | D174,D185,H251,E284,E315 | 3 | 1866770 | 1868163 |
| BBOV_III002280 | XP_001611362 | methionine aminopeptidase, type II family protein | M24A | MG | n/d | n/d | 432 | 48.19 | 5.78 | 109-432 | H184 | D204,D215,H284,E318,E413 | 3 | 534747 | 536152 |
| BBOV_IV006760 | XP_001610600 | methionine aminopeptidase, putative | M24A | MG | MER181329 | methionyl aminopeptidase 1 | 376 | 41.66 | 7.27 | 119-375 | H194 | D211,D222,H285,E318,E349 | 4 | 652995 | 654760 |
| BBOV_IV001950 | XP_001609360 | methionine aminopeptidase I, putative | M24A | MG | MER165750 | methionyl aminopeptidase 1 | 611 | 69.38 | 7.43 | 185-603 | H258 | D275,D286,H514,E546,E578 | 4 | 456764 | 458735 |
| BBOV_III008370 | XP_001611965 | metallopeptidase M24 family protein | M24B | MG | n/d | n/d | 624 | 70.28 | 5.61 | 310-573 | H390,H479,H492 | D409,D420,H483,E523,E537 | 3 | 1804609 | 1806620 |
| BBOV_III005230 | XP_001611654 | ATP-dependent metalloprotease FtsH family protein | M41 | MA | MER181398 | unassigned | 797 | 88.16 | 7.37 | 514-787 | E568 | H567,H571,D643 | 3 | 1111071 | 1113499 |
| BBOV_IV011870 | XP_001611107 | cell division protein metalloprotease FtsH, putative | M41 | MA | MER190621 | i-AAA peptidase | 658 | 72.4 | 8.6 | 427-630 | E479 | H478,H482,D557 | 4 | 1734070 | 1736046 |
| BBOV_II000870 | XP_001609615 | ATP-dependent metalloprotease FtsH family protein | M41 | MA | MER179920 | i-AAA peptidase | 706 | 78.44 | 8.71 | 441-706 | E493 | H492,H496,D569 | 2 | 225488 | 227711 |
| BBOV_IV000310 | XP_001609199 | CAAX metallo endopeptidase, putative | M48A | MA | MER165646 | unassigned | 448 | 52.65 | 8.63 | 174-448 | E307 | H306,H310,E385 | 4 | 80954 | 82378 |
| BBOV_II007480 | XP_001610266 | 26S proteasome regulatory subunit, putative | M67A | MP | MER181245 | Poh1 peptidase | 312 | 35 | 6.59 | 15-311 | E55 | H116,H118,D129 | 2 | 1670576 | 1671662 |
| BBOV_IV004330 | XP_001610362 | DegP protease, putative | S01B | PA | n/d | n/d | 536 | 59.91 | 6.25 | 96-249 | H111,D142,S220 |  | 4 | 128989 | 130811 |
| BBOV_II006080 | XP_001610126 | subtilisin-like protein, putative | S08A | SB | n/d | n/d | 694 | 77.21 | 8.97 | 323-663 | D364,H413,N505,S610 |  | 2 | 1373827 | 1375914 |
| BBOV_II002340 | XP_001609758 | hypothetical protein | S09A | SC | MER144868 | unassigned | 487 | 54.62 | 7.94 | 245-483 | S343,D427,H458 |  | 2 | 573829 | 575464 |
| BBOV_III007870 | XP_001611915 | conserved hypothetical protein | S09X | SC | MER208649 | unassigned | 237 | 26.97 | 5.78 | 5-224 | S64,D133,H206 |  | 3 | 1707764 | 1708576 |
| BBOV_III006090 | XP_001611738 | hypothetical protein | S09X | SC | n/d | n/d | 420 | 46.43 | 5.5 | 27-250 | S141,D215,H245 |  | 3 | 1314753 | 1316197 |
| BBOV_IV005690 | XP_001610498 | hypothetical protein | S09X | SC | MER210788 | unassigned | 215 | 23.86 | 7.19 | 19-196 | S85,D150,H178 |  | 4 | 423381 | 424177 |
| BBOV_II004070 | XP_001609930 | ATP-dependent Clp protease proteolytic subunit 1, putative | S14 | SK | MER192985 | PFC0310c peptidase | 225 | 25.44 | 6.82 | 3-212 | S117,H142,D191 |  | 2 | 944830 | 945580 |
| BBOV_III006020 | XP_001611731 | ATP-dependent protease La family protein | S16 | SJ | MER109626 | PIM1 peptidase | 1122 | 123.52 | 5.77 | 951-1106 | S1028,K1071 |  | 3 | 1295572 | 1298940 |
| BBOV_I004260 | XP_001609075 | conserved hypothetical protein | S26A | SF | n/d | n/d | 152 | 16.69 | 9.37 | 28-143 | S40,K91 |  | 1 | 622747 | 623355 |
| BBOV_III000270 | XP_001611162 | signal peptidase, putative | S26B | SF | MER233718 | unassigned | 183 | 20.55 | 8.54 | 52-183 | S63,H101 |  | 3 | 60388 | 61366 |
| BBOV_II006840 | XP_001610202 | alpha/beta hydrolase protein, putative | S33 | SC | n/d | n/d | 348 | 38.52 | 7.67 | 1-262 | S81,D203,H231 |  | 2 | 1539864 | 1540964 |
| BBOV_II006100 | XP_001610128 | rhomboid 4 | S54 | ST | n/d | n/d | 314 | 35.59 | 9.35 | 2-162 | S40,H92 |  | 2 | 1378723 | 1379879 |
| BBOV_II006070 | XP_001610125 | conserved hypothetical protein | S54 | ST | n/d | n/d | 406 | 46.7 | 9.08 | 80-297 | S169,H220 |  | 2 | 1370986 | 1372581 |
| BBOV_II005950 | XP_001610113 | rhomboid 4 | S54 | ST | n/d | n/d | 629 | 69.69 | 9.46 | 308-486 | S399,H450 |  | 2 | 1349101 | 1351168 |
| BBOV_II005940 | XP_001610112 | rhomboid 4 | S54 | ST | MER112671 | unassigned | 641 | 71.07 | 9.42 | 308-486 | S399,H450 |  | 2 | 1346426 | 1348423 |
| BBOV_II005930 | XP_001610111 | rhomboid 4 | S54 | ST | n/d | n/d | 783 | 85.98 | 9.59 | 461-687 | S552,H603 |  | 2 | 1343175 | 1345678 |
| BBOV_III000530 | XP_001611188 | rhomboid family protein | S54 | ST | n/d | n/d | 496 | 55.55 | 9.7 | 224-496 | S431-H482 |  | 3 | 126070 | 127767 |
| BBOV_I003700 | XP_001609020 | hypothetical protein | S54 | ST | n/d | n/d | 452 | 50.99 | 9.5 | 316-422 | S344,H405 |  | 1 | 498074 | 499468 |
| BBOV_II002710 | XP_001609795 | proteasome A-type and B-type family protein | T01A | PB | n/d | n/d | 217 | 24.18 | 6.37 | 16-217 | T16 |  | 2 | 647543 | 649749 |
| BBOV_II002330 | XP_001609757 | proteasome subunit alpha type 6 protein, putative | T01A | PB | n/d | n/d | 276 | 30.62 | 5.4 | 38-272 | T38 |  | 2 | 571116 | 572263 |
| BBOV_II005970 | XP_001610115 | proteosome A, putative | T01A | PB | n/d | n/d | 266 | 29 | 5.33 | 38-262 | T38 |  | 2 | 1353243 | 1354043 |
| BBOV_IV000320 | XP_001609200 | proteasome subunit alpha type 1 | T01X | PB | n/d | n/d | 257 | 28.88 | 7.6 | 27-235 | T33 |  | 4 | 82583 | 83498 |
| BBOV_IV008660 | XP_001610788 | proteasome subunit beta 7, putative | T01A | PB | MER122074 | proteasome catalytic subunit 2 | 271 | 29.36 | 7.72 | 41-235 | T41 |  | 4 | 1052890 | 1053967 |
| BBOV_IV009000 | XP_001610822 | proteasome epsilon subunit , putative | T01A | PB | n/d | n/d | 268 | 29.88 | 6.26 | 57-261 | T57 |  | 4 | 1118118 | 1119416 |

Table S2. New putative peptidases predicted using the MEROPS database.

| Type | Gene name | Protein Id | Family & subfamily | Clan |
| --- | --- | --- | --- | --- |
| Cysteine | BBOV_I000200 | XP_001608682 | C13 | CD |
|  | BBOV_III006180 | XP_001611747 | C19 | CA |
|  | BBOV_III001650 | XP_001611300 | C19 | CA |
|  | BBOV_IV004350 | XP_001610364 | C19 | CA |
|  | BBOV_IV001730 | XP_001609338 | C19 | CA |
|  | BBOV_II005340 | XP_001610053 | C26 | PC |
|  | BBOV_II005540 | XP_001610073 | C54 | CA |
| Metallo | BBOV_III008650 | XP_001611993 | M24A | MG |
|  | BBOV_III002280 | XP_001611362 | M24A | MG |
|  | BBOV_III008370 | XP_001611965 | M24B | MG |
| Serine | BBOV_IV004330 | XP_001610362 | S01B | PA |
|  | BBOV_II006080 | XP_001610126 | S08A | SB |
|  | BBOV_III006090 | XP_001611738 | S09X | SC |
|  | BBOV_I004260 | XP_001609075 | S26A | SF |
|  | BBOV_II006840 | XP_001610202 | S33 | SC |
|  | BBOV_II006100 | XP_001610128 | S54 | ST |
|  | BBOV_II006070 | XP_001610125 | S54 | ST |
|  | BBOV_II005950 | XP_001610113 | S54 | ST |
|  | BBOV_II005930 | XP_001610111 | S54 | ST |
|  | BBOV_III000530 | XP_001611188 | S54 | ST |
|  | BBOV_I003700 | XP_001609020 | S54 | ST |
| Threonine | BBOV_II002710 | XP_001609795 | T01A | PB |
|  | BBOV_II002330 | XP_001609757 | T01A | PB |
|  | BBOV_II005970 | XP_001610115 | T01A | PB |
|  | BBOV_IV000320 | XP_001609200 | T01X | PB |
|  | BBOV_IV009000 | XP_001610822 | T01A | PB |

**. Cream: cysteine, red: metallo; yellow: serine and orange: threonine.**

Table S2. Comparison between Apicomplexa cysteine peptidases

| Peptidase family | *B bovis* | *P falciparum* | *T gondii* |
| --- | --- | --- | --- |
| C1 | 4 | 9 | 4 |
| C02 | 1 | 0 | 0 |
| C11 | 0 | 0 | 0 |
| C12 | 1 | 2 | 2 |
| C13 | 1 | 1 | 1 |
| C14 | 0 | 1 | 2 |
| C15 | 0 | 0 | 1 |
| C19 | 5 | 7 | 1 |
| C26 | 2 | 1 | 1 |
| C44 | 1 | 1 | 0 |
| C48 | 1 | 1 | 3 |
| C50 | 0 | 0 | 1 |
| C54 | 1 | 0 | 0 |
| C65 | 0 | 0 | 1 |
| C78 | 1 | 0 | 1 |
| C85 | 0 | 1 | 1 |
| C86 | 0 | 1 | 1 |
| C88 | 0 | 0 | 1 |
| Total cysteine peptidases | 18 | 25 | 21 |

Table S4. Specific primers for amplification of *B. bovis* peptidase encoding genes.

| Gene name | Primer sequences (5’ to 3’) | Amplicon size (bp) | Tm (°C) |
| --- | --- | --- | --- |
| BBOV_II005930 | F:AATATTGGCTGCCCGTAACATGGTGGAAG | 474 | 62.4 |
|  | R:ATCACTGATCGGGATTCACGAGAGAATCTC |  | 60.7 |
| BBOV_II006070 | F:GACGGTATCAAGTGCCTTCACACAAAC | 270 | 59.9 |
|  | R:TTGTGCGAAAGACAATCTTTGTGAAACG |  | 58.5 |
| BBOV_III009030 | F:TGTTGTCCAGTGGATTCTGTCAATATATAC | 270 | 56.4 |
|  | R:CGGTACCACATTCGGTAAAATATTTCCAG |  | 57.6 |
| BBOV_III000530 | F:ATGTGCAAATGCTGCGCTGTG | 1488 | 59.6 |
|  | R:AAAAGCCATGGTGAGGCACATTCC |  | 60.3 |
| BBOV_IV011550 | F:ATGTCGCTCAAGGAATCCTC | 1594 | 54.5 |
|  | R:ACAGACGGATCCACTTACG |  | 54.0 |

Table S5. Specific primers for *Babesia bovis* peptidase transcripts analyzed for differential expression between the virulent and attenuated strain pair.

| Gene name^1^ | Primer sequences (5’ to 3’) | Amplicon size (bp) | Tm (°C) |
| --- | --- | --- | --- |
| BBOV_I000200 | F’:CTTTGGGTTTCTTTGGCAGG | 124 | 65.5 |
|  | R’:CATGTCCCGCTCTATACCATG |  | 64.1 |
| BBOV_I000540 | F’:TCTATCCCCTTATCGTTTTCTG | 197 | 60.8 |
|  | R’:TGGCGAGGTAGAGCTAAATTG |  | 63.0 |
| BBOV_II001130 | F’: AGCGGATGTAGTTGAAGCAC | 110 | 61.8 |
|  | R’:TTCTTGCGATATTCCAGGGTG |  | 65.8 |
| BBOV_III003510 | F’:CCGCCAGAAAAGTTTGTAGTG | 141 | 63.4 |
|  | R’:AGTACGCATTCACCAGTTCC |  | 61.6 |
| BBOV_I004260 | F’:TTGTCACCTTCTAACCACACG | 155 | 62.5 |
|  | R’:CAGTCCCGTTCCAATATAC |  | 56.8 |
| BBOV_III000270 | F’:ATGGACTACATCAAATCGGAAATTAAC | 119 | 64.6 |
|  | R’:CAGGGCTGTGAAGAGTACG |  | 61.6 |
| BBOV_IV008660 | F’:TGGCGTGTTGGTAAAGGATG | 102 | 65.7 |
|  | R’:TGATATACGGTGTAACTTGGAGC |  | 61.6 |
| BBOV_III004820 | F’:GGGATAGGTACAGGATTCAGC | 197 | 61.3 |
|  | R’:TCCGTTTTCATCTAGCCACTG |  | 64.0 |

^1^ Genes in blue and black fonts are those that were upregulated in T2Bo_att and T2Bo_vir, respectively. BBOV_III004820 is the gene for normalization (green font).
