# Supplementary material for: Genome-wide analysis of peptidase content and expression in a virulent and attenuated Babesia bovis strain pair
Source: Mol Biochem Parasitol. 2011 Oct;179(2-2):111–3. doi: 10.1016/j.molbiopara.2011.06.005 (PMC3167272; doi:10.1016/j.molbiopara.2011.06.005)
Supplement: Supplementary file 2 [file mmc2.zip › mmc2.pptx]

## Slide 1
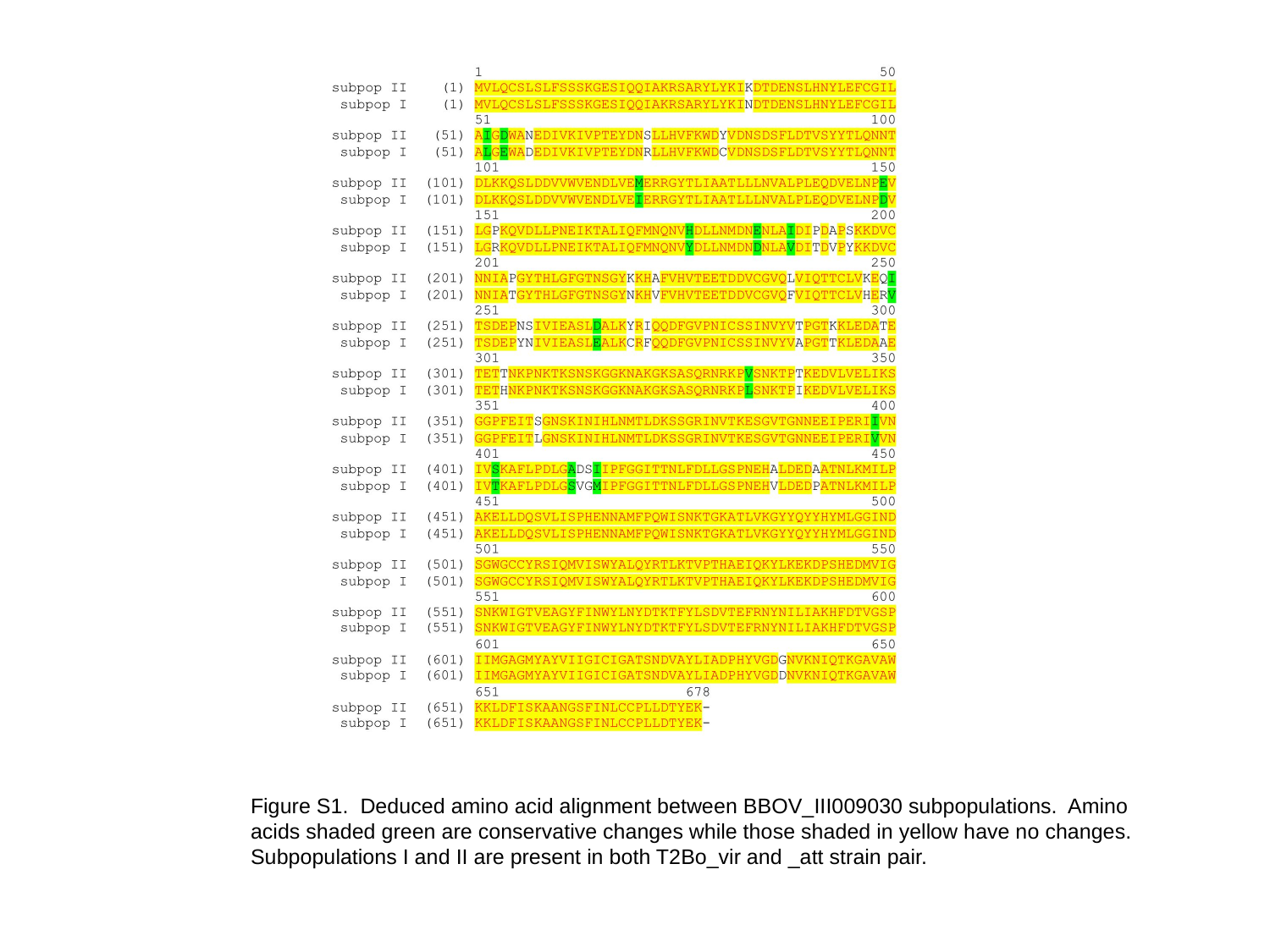

Figure S1. Deduced amino acid alignment between BBOV_III009030 subpopulations. Amino
acids shaded green are conservative changes while those shaded in yellow have no changes.
Subpopulations I and II are present in both T2Bo_vir and _att strain pair.

## Slide 2
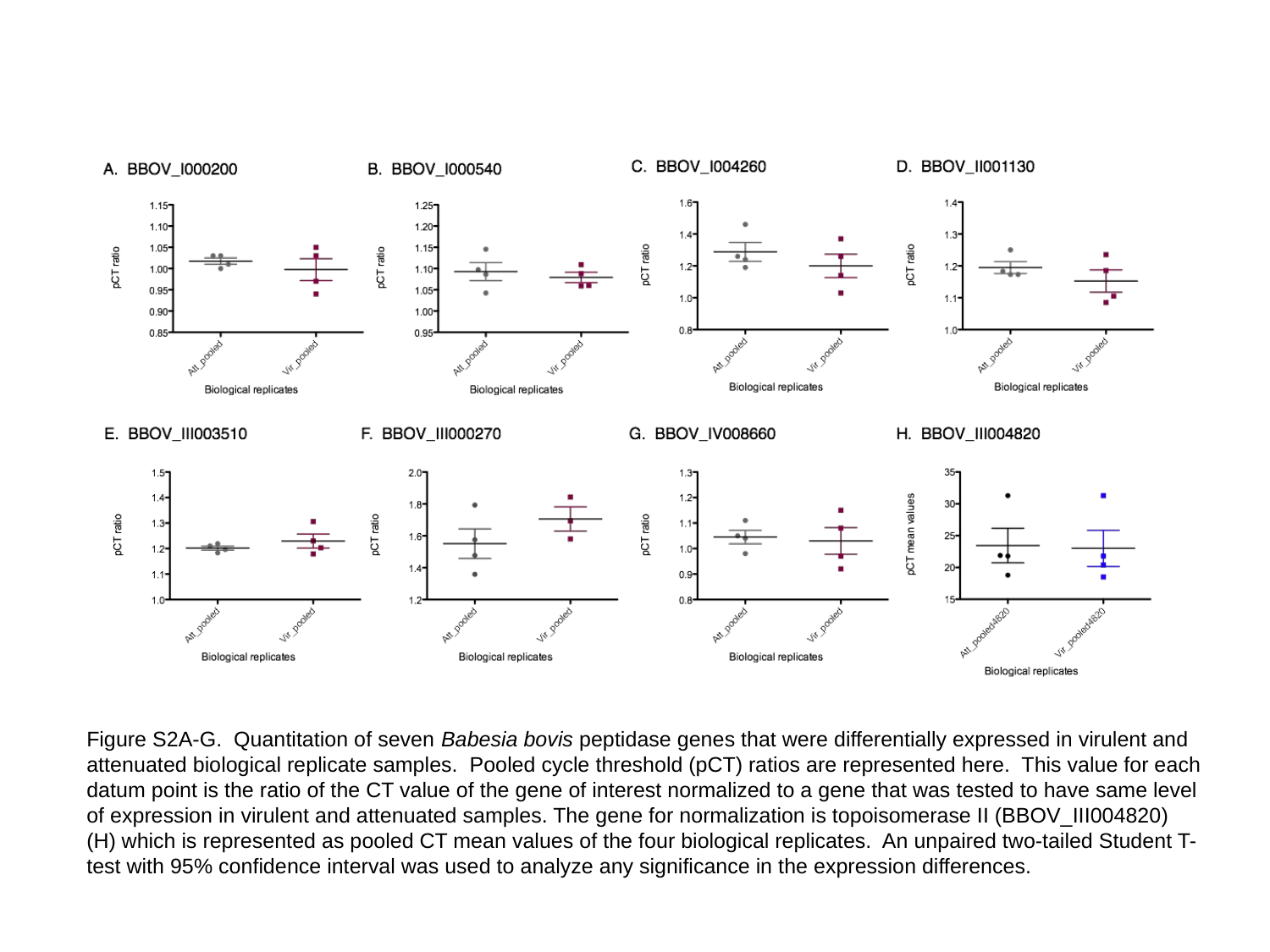

Figure S2A-G. Quantitation of seven Babesia bovis peptidase genes that were differentially expressed in virulent and attenuated biological replicate samples. Pooled cycle threshold (pCT) ratios are represented here. This value for each datum point is the ratio of the CT value of the gene of interest normalized to a gene that was tested to have same level of expression in virulent and attenuated samples. The gene for normalization is topoisomerase II (BBOV_III004820) (H) which is represented as pooled CT mean values of the four biological replicates. An unpaired two-tailed Student T-test with 95% confidence interval was used to analyze any significance in the expression differences.
