# Supplementary material for: Genome-wide analysis of peptidase content and expression in a virulent and attenuated Babesia bovis strain pair
Source: Mol Biochem Parasitol. 2011 Oct;179(2-2):111–3. doi: 10.1016/j.molbiopara.2011.06.005 (PMC3167272; doi:10.1016/j.molbiopara.2011.06.005)
Supplement: Supplementary file 3 [file mmc3.doc]

**Supplemental Material and Methods:**

*Whole genome transcriptome array design and statistical analyses:*

A single 45 bp gene-specific oligonucleotide probe was designed for each of the 66 putative *B. bovis* peptidase transcripts. These were then spotted onto glass microscope slides according to specification of MYcroarray.com. Each probe was randomly placed at five locations on each array. Three arrays per biological replicate were included as technical replicates. Since each peptidase gene was represented by five copies of a specific probe per chip, the transcription of every peptidase gene was individually assessed 45 times in each strain. Internal chip controls, provided by MYcroarray Inc. were placed along with the specific *B. bovis* peptidase oligonucleotide probes and were used to evaluate background intensities for each array.

Data was extracted from the scanned images using GenePix Pro software (version 6.1.0.4); and a median feature pixel was calculated. Flagged, saturated features and background correction were all evaluated as the trimmed mean was used to estimate the signal for each probe set. Normalization was done to adjust for differences in dye incorporation. Briefly, a scale factor was created to equalize the signal across all arrays. Only features that met the following criteria were called normalization features (NF) and were used to calculate the scale factor: (i) <10% saturation pixels; (ii) median signal 6 times higher than the background; and (iii) presence in all arrays. The average signal for the normalization features was calculated for each array. A scale factor (SF) for each array was calculated as follows:

SF = Average (NF median signal) array I / Average (NF median signal) Brightest array

A trimmed mean statistic was used to estimate the signal value for each probe set. The median pixel signal for each feature was used after background subtraction and scaling. For each probe set (n=5) the maximum and minimum values were left out and the remaining 3 values were averaged. If there were less than 5 probe signal values due to either a bad or saturated feature, the trimmed mean was simply calculated on the remaining probes.

An estimation of whether or not a transcript was detected by a probe was calculated. The estimation is a “present” call. The “present” call is based on the signal value for each gene (probe) relative to the local median background level surrounding each spot. For each probe, the transcript was considered “present” if the signal was 5 times higher than the background signal.

The global *Babesia bovis* peptidase gene expression profile was generated in the following manner. Each biological replicate pair, T2Bo_vir and _att group, was compared separately. The filtering criteria were as follows: (1) For each gene, at least 4 out of 5 probes had to have a “present” call for at least one condition (either “A” or “V”). (2) For the genes that satisfied the present call filter (#1 above), a Student’s T-test was performed on the Log_2_ transformed signal data. Only genes with at least p<0.05 were considered. (3) Finally, a gene was called differentially expressed if it showed a change ratio (A/V was used arbitrarily) of at least 1.5-fold combined with a T-test p<0.01.

*Quantitative PCR analysis:*

Quantitation of transcript expression by measurement of the cycle threshold (CT) levels of seven genes was performed (Table S5). The selection criterion for these genes is based on the A/V (Att/Vir) ratio obtained from the microarray data for any of the biological replicate pairs that were >2 or <0.5, specifically those genes that were up-regulated by at least two-fold in the attenuated or virulent *B. bovis*, respectively, in at least one of the three microarray replicates. As there were differences among the three replicates, an independently prepared biological replicate pair #4 was included in the qPCR analysis along with the original three biological replicate pairs in the validation process. Prior to qPCR, DNase-treated RNA was reverse transcribed using the RETROscript® Kit (ABI) to create cDNA templates in accordance with manufacturer’s protocols. PCR was set up to amplify the seven peptidase genes to be queried with gene-specific primers using the following cycling parameters: initial denaturation at 95°C for 3 min followed by 35 cycles of 95°C for 30 s, 55°C for 30 s, 72°C for 1 min with a final extension of 5 min. Amplicons were analyzed to confirm the specificity of the primers for each gene (Table S5). For qPCR, DNase-treated total RNA was reverse transcribed followed by PCR in the presence of iQ SYBR Green Supermix (BioRad) with the following amplification protocol: initial denaturation at 95°C for 10 min followed by 40 cycles of 95°C for 10 s, 57.5°C for 30 s, 72°C for 30 s followed by a melting curve of 72°C for 5 min, 55°C for 1 min, 55°C for 10 s with 0.5°C increases for 80 cycles. PCR amplification was performed in quadruplicate for each biological replicate sample pair (#1-4). DNase-treated RNA only and water were included as negative controls. All CT raw values were normalized to *B. bovis* topoisomerase II transcript (BBOV_III004820), whose transcript levels are the same in both T2Bo_vir and _att samples (data not shown). The resulting normalized value for each gene is represented as CT ratio. CT ratios obtained by all the biological replicate pairs were subsequently pooled for both virulent and attenuated samples and these pooled ratios were analyzed using an unpaired two-tailed Student T-test with confidence interval set at 95% (GraphPad Prism v. 5.0a). Results are shown in Fig. S2.
